# Role of N-acetylcysteine treatment in women with advanced age undergoing IVF/ICSI cycles: A prospective study

**DOI:** 10.3389/fmed.2022.917146

**Published:** 2022-10-04

**Authors:** Xiufang Li, Zhongqing Wang, Huidan Wang, Haiyan Xu, Yan Sheng, Fang Lian

**Affiliations:** ^1^The First Clinical College, Shandong University of Traditional Chinese Medicine, Jinan, China; ^2^Center for Reproductive Medicine, Shandong University, Jinan, China; ^3^Medical College of Optometry and Ophthalmology, Shandong University of Traditional Chinese Medicine, Jinan, China; ^4^Department of Traditional Chinese Medicine, Jinan Golden Time Health Nursing Hospital, Jinan, China; ^5^Integrative Medicine Research Centre of Reproduction and Heredity, Affiliated Hospital of Shandong University of Traditional Chinese Medicine, Jinan, China

**Keywords:** N-acetylcysteine, advanced age, GSH, mtDNA copy number, blastocyst, pregnancy outcome

## Abstract

**Objective:**

The main objective of this study was to explore the efficacy of a new antioxidant N-acetylcysteine (NAC) supplementation in reproductive outcomes of advanced age women undergoing *in vitro* fertilization/intracytoplasmic sperm injection-embryo transfer (IVF/ICSI-ET), and the effect on the expression of L-glutathione (GSH) in follicular fluid (FF) and mitochondrial DNA (mtDNA) copy number of granulosa cells.

**Methods:**

The present prospective randomized controlled study was conducted in 200 patients with advanced age women undergoing GnRH antagonist protocol. The treatment group (group A) consisted of 100 women who received N-acetylcysteine treatment from the menstrual phase of the previous cycle for about 45 days using the GnRH antagonist protocol. The control group (group B) consisted of 100 women who received the same protocol without N-acetylcysteine. Total gonadotrophin dosage the number of oocyte received, high-quality blastocysts, and pregnancy outcomes were compared between two groups. Pregnancy outcomes included biochemical pregnancy rate, clinical pregnancy rate, embryo implantation rate, ectopic pregnancy rate, multiple pregnancy rate, and ongoing pregnancy rate. Follicular fluid (FF) was collected after oocytes were gathered. The GSH content in the FF was tested with enzyme linked immunosorbent assay (ELISA). The mtDNA copy number of the granulosa cells was measured using real-time PCR techniques.

**Results:**

Total doses of Gn in the NAC treatment group were less than those in the control group (2385.50 ± 879.19 vs. 2527.63 ± 1170.33, *P* = 0.047). Compared with the control, the number of high-quality blastocysts in NAC treatment increased significantly (1.82 ± 2.12 vs. 1.43 ± 1.58, *p* = 0.014). Clinical pregnancy rates did not differ in both groups (all *P* > 0.05). At the same time, the GSH content in the FF differed significantly between the two groups (1.88 ± 1.23 vs. 1.07 ± 0.70, *p* = 0.001). There was no significant difference in the mtDNA copy number between the two groups (*P* = 0.157).

**Conclusion:**

A combination of NAC and Gn treatment is capable of improving the ovarian response to superovulation drugs in assisted reproductive technologies (ARTs) and also in aged populations. The addition of NAC during IVF can improve the quality of blastocysts in advanced age female subjects. However, more clinical trials are required to be designed to confirm this conclusion in future.

**Ethics and dissemination:**

The experiment solicited approval from the Institutional ethics committee of the Affiliated Reproductive Hospital of Shandong University. All the participants provided written informed consent. This survey was conducted as per the Declaration of Helsinki and relevant amendments.

**Trial registration number:**

www.chictr.org.cn, identifier ChiCTR2100048297.

## Introduction

The success of pregnancy in humans is closely related to the age at which women want to conceive all over the word (1, 2). Since the officially implementation of the “two-child policy” in 2016, ~90 million Chinese women have been eligible to have a second child. However, 60% of these potential second-child mothers are advanced maternal age women whose age is above 35, and more than half are 40 years older (3, 4), and those elder female subjects with reduced fertility usually ask for assistance from assisted reproductive technologies (ARTs). Correspondingly, the number of women over 35 years old undergoing *in vitro* fertilization /intracytoplasmic sperm injection (IVF/ICSI) treatments is growing rapidly worldwide (5–7). In ART, age is the most critical predictor of success (8). The main causes of age-related infertility are that as people get older, their ovarian reserve and oocyte/embryo competency decline reduced. Fertility declines with the woman aging, while the incidence of pregnancy loss follows the opposite direction. Oocyte and embryo quality is a key factor for pregnancy success and declines with age. At present, various strategies have been conducted to promote the ovarian response and increase the outcome of clinical pregnancy in advanced age patients who undergo IVF/ICSI, including the growth hormone (GH) (9), dehydroepiandrosterone (DHEA) (10), recombinant luteinising hormone (r-LH) (11), and coenzyme (12). However, it is controversial that those drugs can increase pregnancy outcomes in advanced age women.

Oocyte aging is closely related to mitochondrial dysfunction and oxidative stress. The ability of dysfunctional mitochondria to resist oxygen species (ROS) production which leads to oxidative stress is lower. The mtDNA copy number rapidly increases during oocyte maturation. Thus, the availability of an adequate number of functional mitochondria is very important. The mtDNA copy number was decreased dramatically in patients with diminished ovarian reserve compared to female subjects with normal ovarian reserve (13). The mitochondrial function might be improved with the application of antioxidants. Oxidative stress was regarded as a causative factor for aging, which was also a prominent mediator associated with oocyte damage and causes poor embryonic development (14, 15). N-acetylcysteine (NAC), a precursor of reduced glutathione (GSH), is a well-known antioxidant. Several studies have reported that the antioxidant activity of NAC is by using different substrates, methods, and oxidants to assess the oxidative processes *in vitro* (16, 17). So NAC has been used as an antioxidant in several *in vivo* studies (18). NAC could scour free radicals and improve oocyte quality in aged mice during oocyte cryopreservation (18). However, studies regarding NAC efficacy in patients with advanced age are rare. The influence of mitochondrial function on quality of oocytes is significant. Our research attempted to investigate the effect of NAC on the quality of oocytes, embryos, and pregnancy outcomes in advanced patients undergoing IVF/ICSI cycles. We also performed the analysis of GSH and mtDNA copy numbers after oral NAC in infertile aged women.

## Materials and methods

### Study population

The current single-blinded clinical experiment was conducted among 200 advanced age patients who visited the Affiliated Reproductive Hospital of Shandong University for their infertility treatment from August 2021 to January 2022 after institutional ethics clearance. These patients were first evaluated and then randomized into two groups. The experimental procedures were in accordance with the Helsinki Declaration. The current research is documented in the Chinese Clinical Trial Registry (ChiCTR2100048297).

**Figure 1:**
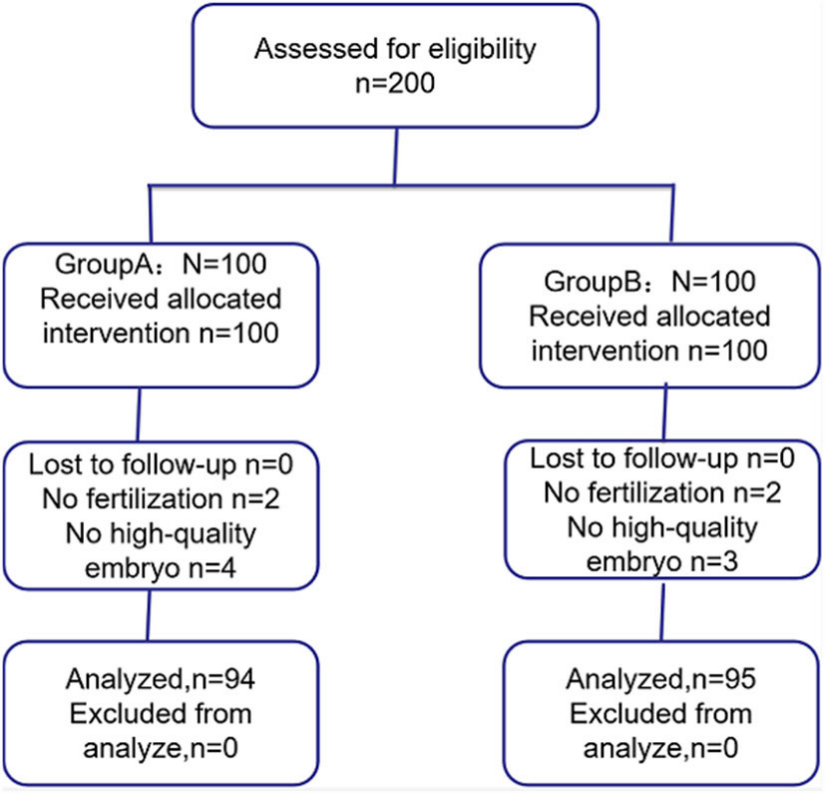
CONSORT flow diagram for progress of the participants of the randomized trial.

Of the 200 patients, six patients from group A (two patients failed fertilization and four patients failed high-quality embryo) and five patients from group B (two patients failed fertilization and three patients failed high-quality embryo) were not included (Figure 1). Finally, 94 patients from group A and 95 patients from group B were included in this study.

### Study interventions

According to a computer-generated randomization list, 100 patients were randomized into the NAC group to receive NAC 0.6 g three times daily from the menstrual phase prior to the IVF/ICSI cycles for nearly 45 days. Moreover, 100 patients were randomized into the control group to directly enter the IVF/ICS cycles. All patients received the antagonist protocol. After injecting patients with 150–300 IU of gonadotrophin to stimulate ovulation, the dose of GnRH-a was 0.25 mg/day when the diameter of the dominant follicle was ≥11–12 mm or LH > 10 IU/L (or >two times the baseline level) or E2 > 200–300 pg/ml. If the maximal follicular diameter was 14 mm, 0.25 mg of the GnRH antagonist cetrorelix was given subcutaneously every day until late ovarian stimulation. If more than one mature follicle of a diameter ≥18 mm could be detected through ultrasound, ovulation was triggered through an 8,000-IU intramuscular injection of human chorionic gonadotropin (HCG). Oocyte puncture received 36 h HCG application through guided transvaginal ultrasonography. Samples were carefully collected as a follicular puncture. Follicular fluid was collected from the follicle of women undergoing IVF-ET treatment to test for GSH and mtDNA.

Normally, two embryos were transplanted on day 3 following the puncture. If the patient's uterus is deformed, such as scar uterus, single horn uterus, or cervical incompetence, one embryo was transferred on day 3 or day 5 to reduce the risk of miscarriage. Moreover, the remaining embryos were incubated. The embryos were frozen if blastocysts formed. Luteal phase support was initiated on the date of oocyte puncture, and all patients were administered with 20 mg dydrogesterone (Abbott, the Netherlands) two times daily and 200 mg utrogestan vaginal suppositories (Laboratoires Besins International, France). Serum beta HCG levels were evaluated 12–14 days following embryo transfer for the confirmation of biochemical pregnancy. Progestin support lasted until the late 12-weeks gestation period after a successful pregnancy. Clinical pregnancy was defined as the detection of an intrauterine gestation alsac by transvaginal ultrasonography after 6 weeks of embryo transfer. Ongoing pregnancy was defined as the presence of a fetus with heart motion at 12 weeks of gestation. The ongoing pregnancy rate, number of gathered oocytes, fertilized oocytes, and frozen embryos, clinical pregnancy rate, implantation rate, multiple pregnancy rate, pregnancy loss rate, and ectopic pregnancy rate were all used to assess embryo transfer.

### Study outcomes

The primary outcome measure was the number of high-quality blastocysts and GSH content in follicular fluid in advanced age patients. Fertilization rate, clinical pregnancy rate, embryo implantation rate, multiple pregnancy rate, miscarriage rate, ectopic pregnancy rate, and ongoing pregnancy are secondary outcomes.

### Embryo grade

Embryos were graded from one to four on the third day of incubation, based on percent cell counts and fragmentation, as follows: grade 4, equal-sized symmetrical cells with no fragmentation; grade 3, equal-sized symmetrical cells with 1/3 fragmentation; grade 2, non-symmetrical blastomeres with 1/3–1/2 fragmentation; and grade 1, only one or two symmetrical cells with abundant of fragmentation (19). In our center, embryos standard ≥ grade 3 are defined as excellent embryos.

Gardner grading criteria were used to divide blastocysts into six periods based on blastocoel formation. Stage 1: Early cavitary blastocyst with blastocyst cavity <1/2 of the embryo volume; Stage 2: blastocyst cavity coelom greater than or equal to 1/2 of the volume; Stage 3: fully expanded blastocyst, blastocyst cavity occupies the embryo; Stage 4: expanded blastocyst, blastocyst cavity volume greater than an early embryo, zona thinning; Stage 5: blastocyst being hatched, trophoblast begins to break through the zona pellucida; Stage 6: hatched blastocyst, the blastocyst is completely hatched from the zona pellucida. Stage 3–6 blastocysts need to score the inner cell mass and trophectoderm cells, and the inner cell mass is scored, A: the inner cell mass is tight and the number of cells is large; B: the inner cell mass is loose and the number is small; C: the number of the inner cell mass is very small; the score of the trophectoderm, A: the number of cells is large and a tightly arranged cell layer is formed; B: the number of cells is small and the arrangement is loose; C: the trophectoderm is composed of sparse cells. In our center, blastocysts with recovered blastocyst standard ≥ stage 4 (4BC) and blastocysts with score ≥ stage 4 (4BC) are defined as high-quality blastocysts.

The embryos transferred in IVF/ICSI cycles of all patients in two groups are high-quality embryos on day 3 or day 5.

### Determination of GSH in the follicular fluid by ELISA

The GSH content in FF was measured with the ELISA Kit (Jiang's biological, Shanghai, China) by enzyme-linked immunoassay technique, following the manufacturer's instructions. The correct use of the reagent was to make serial dilutions, i.e., 20, 10, 5, 2.5, 1.25, 0.625 umol/L. The follicular fluid samples were defrosted and then mixed together and 50 μl of FF was added to each well in duplicate. The absorbance value was measured at 450 nm with a Tecan infinite F50 microplate spectrophotometer (Longyue Biological Technology Development Co., Ltd., Beijing, China). Such samples were diluted two times, and 50 μl of diluted FF was then added to each well.

### Granulosa cells isolated from follicular fluid

Follicular fluids were centrifuged at 1,500 r/min for 5 min, and the supernatant was discarded. The deposition (containing grains cells and blood cells) in 50% Percoll 2 ml was centrifuged at 4,000 r/min for 10 min to separate granulosa cells and red blood cells. To obtain clean granulosa cells, the isolated granulosa cells were washed two times with PBS and then placed in 50% Percoll to remove the flocculent.

### DNA extraction from granulosa cells

The DNA in granulosa cells was extracted in lysis buffer (Yeasen Biotech Co., Ltd., Shanghai, China) by heating at 55°C for 30 min, followed by 95°C for 3 min. To extract the DNA present from the original medium, the culture medium was transferred to a microtubes, and cells were removed by centrifugation at 4,000 × *g* for 1 min, and the spent culture medium was mixed with the same amount of lysis buffer and followed the steps above.

### Evaluation of mtDNA copy numbers from granulosa cells

mtDNA copy number was determined by real-time PCR using mitochondrial DNA probe fluorescent quantitative PCR reagent (RayzeBio Technology Co., Ltd., Shanghai, China). The samples were continuously diluted in 10-fold order to construct PCR quantitative standard curve. The real-time PCR instruments (Hehui Biotechnology) meet the following conditions: one cycle at 95 °C for 3 min, followed by 45 cycles of 95 °C for 15 s, and 60 °C for 60 s. The absolute mtDNA copy number in granulosa cells of each sample was calculated using a standard curve derived from PCR amplifications by 2^−ΔΔCT^ method.

### Statistical analysis

According to the previous literature, the average number of high-quality embryos cultured in advanced age patients was 1.27 ± 0.28 (20). Due to the lack of data on the number of oocytes retrieved after taking NAC in patients with advanced aged women, it is hypothesized that the number of oocytes retrieved after taking NAC can increase by 1. Using the sample size calculation software PASS 11.0 (NCSS, LLC. Kaysville, Utah, USA), a power of 0.9 and a significance level of 0.05 were set to include 178 participants. Assuming a 10% dropout rate, a total of 200 patients were required in both groups, with 100 patients in each group.

Continuous variables can be indicated by mean ± standard deviation (SD), whereas the intergroup difference between these variables was compared using Student's *t*-test. Qualitative variables are presented as the proportion and were interpreted using the χ^2^ test. SPSS version 22.0 was applied in statistical analysis, and a *P*-value of < 0.05 denoted statistical significance.

## Results

Differences between treatment and control groups in terms of the baseline features, such as mean age, body mass index (BMI), primary infertility, number of IVF procedures, AMH levels, follicle-stimulating hormone (FSH) levels, leutenising hormone (LH) levels, and T hormone levels, were statistically nonsignificant. Intergroup differences in the primary infertility ratio and fertilization type were also statistically nonsignificant (Table 1)

**Table 1:** Baseline characteristics of the study population^*^.

**Index**	**Group A** **(*n* = 100)**	**Group B** **(*n* = 100)**	***P*** **value**
Mean age (years)	37.83 ± 2.09	37.81 ± 1.79	0.431
BMI (kg/m^2^)^y^	24.03 ± 2.84	24.08 ± 2.96	0.969
Infertility duration (years)	4.04 ± 3.11	4.03 ± 2.74	0.095
Primary infertility (%)	37 (21)	35 (22)	0.883
Basal FSH (IU/L)	7.90 ± 1.60	7.45 ± 1.71	0.082
Basal LH (IU/L)	5.71 ± 5.21	5.11 ± 4.17	0.551
Basal E2 (pg/mL)	39.37 ± 11.92	40.10 ± 14.41	0.898
Basal T (ng/dL)	20.86 ± 10.33	21.42 ± 12.38	0.593
AMH (ng/mL)	1.93 ± 1.17	2.20 ± 1.08	0.432
TSH (uIU/ml)	2.14 ± 0.91	2.03 ± 0.92	0.576
Antral follicle count	9.77 ± 4.11	10.71 ± 4.29	0.455
Proportion of IVF (%)	74 (74)	74 (74)	1.000
Proportion of ICSI (%)	26 (2)	26 (2)	1.000

* *Plus-minus values can be indicated by means* ± *SD. No significant intergroup differences existed in baseline characteristics (p* > *0.05*). *FSH, follicle-stimulating hormone; LH, luteinising hormone; T, total testosterone; E2, oestradiol; AMH, anti-Müllerian hormone; IVF, in vitro fertilization; ICSI, intracytoplasmic sperm injection*.

*BMI, body mass index can be calculated as the weight (kilograms) divided by the square of height (meters)*.

*Data are number/total number or number (%) unless stated otherwise*.

Differences between the NAC group and the control group in terms of the baseline features, including mean age, body mass index (BMI), infertility duration, primary infertility, number of IVF procedures, follicle-stimulating hormone (FSH) levels, leutenising hormone (LH) levels, T hormone levels, and thyroid stimulating hormone (TSH) levels were statistically non-significant. Intergroup differences in the primary infertility ratio and fertilization type were also statistically non-significant. Moreover, the number of antral follicles in the two groups was matched; thus, their ovarian reserve was comparable (Table 1).

In this clinical trial, fertility outcomes including endometrial thickness, duration of gonadotropin (Gonal-f) treatment, number of retrieved oocytes and transferred embryos, chemical pregnancy, clinical pregnancy rate, implantation rate, early miscarriage rate, ectopic pregnancy rate, and ongoing pregnancy rate were found to vary between two groups, but such differences were statistically non-significant (Table 2). Patients undergoing NAC treatment exhibited a greater number of blastocyst (1.82 ± 2.12) compared to the control group and corresponding differences were statistically significant (1.43 ± 1.58; *P* = 0.014; Table 2). And total doses of Gonal-f were less in patients treated with the NAC group (2385.50 ± 879.19 vs. 2527.63 ± 1170.33, *P* = 0.047; Table 2). After standardized ovarian stimulation, the transplant was canceled for 11 patients, of which four patients exhibited fertilization failure and four patients in the treatment group and three patients in the control group exhibited high-quality embryo failure. No adverse event was observed in the present study.

**Table 2:** Therapeutic outcome in the study population^*^.

**Outcome**	**Group A** **(*n* = 100)**	**Group B** **(*n* = 100)**	***P*** **value**
Endometrial thickness on hCG^y^ trigger day (cm)	1.02 ± 0.18	1.02 ± 0.20	0.445
Duration of gonadotropin (Gonal-f) treatment (d)	9.50 ± 2.44	9.90 ± 2.27	0.893
Total doses of Gonal-f (IU)	2385.50 ± 879.19	2527.63 ± 1170.33	0.047
No. of collected oocytes	6.63 ± 3.55	7.14 ± 3.43	0.604
Fertilization rate (%)	566/663 (85.4)	598/714 (83.8)	0.413
No. of embryos transferred	1.66 ± 0.48	1.64 ± 0.48	0.556
Single embryo transfer^y^ (%)	31 (32.6)	36 (37.9)	0.544
Double embryos transfer^y^ (%)	64 (67.4)	59 (62.1)	0.544
No. of high-quality blastocysts^?^	1.82 ± 2.12	1.43 ± 1.58	0.014
Biochemical pregnancy rate (%)^§^	59 (59)	49 (23)	0.202
Clinical pregnancy rate (%)^||^	51 (24)	43 (25)	0.321
Implantation rate (%)^z^	62/164 (37.8)	51/161 (31.7)	0.294
Multiple pregnancy rate (%)^¶^	13/51 (25.5)	9/43 (20.9)	0.634
Pregnancy loss rate (%)	8 (15.7)	8 (18.6)	0.786
Ectopic pregnancy rate (%)	2 (3.9)	1 (2.3)	1.000
Ongoing pregnancy rate (%)^**^	41 (41.0)	34 (34.0)	0.381

* *Plus-minus values are indicated by means* ± *SD*.

y *hCG, human chorionic gonadotropin*.

^y^*Denominator defined as number of transplants in each group*.

? *The number of high-quality embryos was calculated as the total number of excellent embryos per group and the number of excellent embryos per capita per group. Defined as Gardner score 4BC or higher*.

§ *Biochemical pregnancy, i.e., serum* β*-hCG level* ≥*10 mIU/ml*.

|| *Clinical pregnancy referred to observations about the gestational sac using ultrasonography*.

z *Embryo implantation rate was measured as number of observed intrauterine gestational sacs divided by the number of transferred embryos*.

¶ *Multiple pregnancy rate was calculated as number of multiple pregnancy divided by the number of clinical pregnancy. All the multiple pregnancies are twins*.

** *Ongoing pregnancy suggested the fetal heartbeat detected using ultrasonography until the 12th week of gestation*.

*Data are number/total number or number (%) unless stated otherwise*.

**Figure 2:**
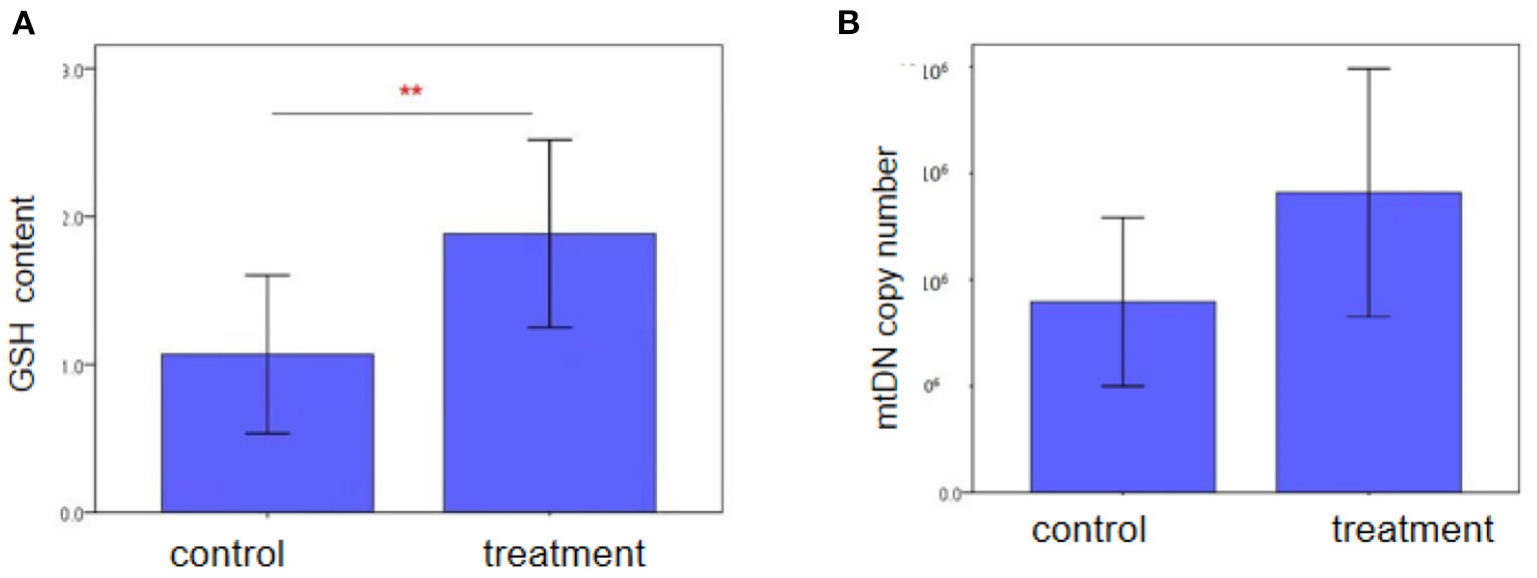
Intracellular GSH content and mtDNA copy number of granulosa cells in advanced women follicular fluid in the control and treatment groups, as **(A)** and **(B)** shown. GSH content in the NAC treatment group was higher than those in the control group (*P* < 0.05). Each treatment was repeated 2 times. The data were analyzed with an independent-samples *T*-test. And mtDNA copy number was similar bwtween two groups (*P* > 0.05).

Characteristics of two groups regarding the GSH content in follicular fluid and mtDNA copy number of granulosa cells were also analyzed between the two groups. As shown in Figure 2, there was a significantly difference in patients who were treated with NAC compared with the control group in the GSH content (1.88 ± 1.23 vs. 1.07 ± 0.70, *P* = 0.001). However, we did not see the differences in the mtDNA copy number of granulosa cells between the two groups.

## Discussion

With increased education and women's career goals, as well as the misperception that ART can compensate for the natural decline in infertility with aging, the proportion of women delaying childbearing has greatly increased, posing a serious challenge for fertility doctors who are facing an increase in the number of women over 35 years old seeking help to conceive. Especially among them over 43, the chance of getting a blastocyst with normal chromosomes is <5% (26–28). The reason was associated with the progressive decline in oocyte/embryo competence in advanced age women, which was the ability to produce a live birth (2, 29, 30). So we aimed to obtain the ideal number of oocytes and culture a larger number of blastocysts in advanced age women according to their specific features by a proper dose of gonadotrophins. In this present prospective randomized controlled study, we did not find differences in clinical pregnancy rates between the two groups. However, compared with the control group, NAC addition significantly increased the number of high-quality blastocysts.

In female subjects over the age of 35, reproductive capacity begins to decline and the rate of miscarriage increases. This decline is primarily due to diminished ovarian reserves and decreased developmental competence of oocytes. The poor quality of oocytes with age is regarded as the main reason for the reduced embryonic developmental competence. The primary cause is oxidative stress (OS) in oocytes. To reduce these adverse effects in oocytes and embryos, various antioxidants had been applied in IVF (31). NAC, the N-acetyl derivative of natural amino acid L-cysteine, for decades has been usually used as a mucolytic (16), and also treats malignancy, atherosclerosis and its complications, and cystic fibrosis (32). In recent years, it has been applied as adjuvant therapy for female infertility as a novel antioxidant (16). Actually, NAC has been used to treat polycystic ovarian syndrome (PCOS) with menstrual cycle disorders and ovulatory dysfunction, and it has been shown that the pregnancy rate in patients with NAC is three times higher than in control patients, with the former having a higher live birth rate (33). Another RCT was conducted by Mostajeran et al. (34) to observe the effect of NAC combined with Letrozole in the treatment of PCOS in 130 women, which reveals a significant increase in sizes of follicles, ovulation rate, and pregnancy rate, and NAC can also improve oocytes quality of PCOS (35). However, the study regarding NAC efficacy in advanced age patients is rare.

The pregnancy rate in IVF/ICSI-ET cycles is closely related to the quality of transplanted embryos and the endometrial receptivity to embryos, especially the former (22, 36, 37). In our study, all transplanted embryos in the present study were high-quality embryos (cleavage embryos or a blastocyst), so pregnancy outcomes including biochemical pregnancy rate, clinical pregnancy rate, implantation rate, and ongoing pregnancy rate between the two groups were similar. However, the number of higher-quality blastocysts was greater among patients receiving IVF cycles along with NAC treatment than those directly undergoing IVF cycles, which can increase the chances of successful clinical pregnancy. Whitaker et al. (38) found that NAC addition to porcine zygotes cultured *in vitro* and during oocyte maturation (21) increased the blastocyst rate compared to the control. Li et al. (39) also found that NAC weakened the bisphenol A-induced downregulation of the fertilization and blastocyst formation rates of mouse *in vitro*. In our present study, total doses of Gonal-f were less in patients treated in the NAC group. Thus, a combination of NAC and Gn treatment is capable of improving the ovarian response to superovulation drugs in ART and also in aged populations. Therefore, NAC is beneficial to follicle development. This finding is concurrent with those of several animal studies, which suggest that NAC could prevent apoptosis and aging to improve the quality of oocytes (18). Another study showed that NAC could improve the quality of Bovine oocytes and embryos by increasing intracellular GSH synthesis, and NAC could be regarded as a preventive antioxidant embryo cultured *in vitro* (15). NAC plays an important role in cell survival through the production of follicular preservation and trophic factor *in vitro* (40, 41). At the same time, our experiments have demonstrated that NAC can improve the clinical rate of subjects, which may be related to the increase in the quality of embryos in advanced age patients. Matilla et al. (42) discovered that NAC supplement after verification could improve the quality of vitrified mature murine oocytes. Fertilization is another significant indicator of the high-quality oocytes. Mukunoki and his colleagues evaluated that NAC could increase the fertilization rate of vitrified-warmed mouse oocytes (43).

The NAC antioxidant potential due to the maximum extent is a precursor of GSH, and glutathione is the primary antioxidant in cells. GSH, which is composed of L-cysteine (L-Cys), glycine (Gly), and L-glutamic acid (L-Glu) residues, plays leading role in regulating cellular processes and mitigating the toxic effects of antioxidants (25, 44). Adding L-cysteine in culture media has been proven to improve the maturation rate of oocytes (45) and increase the developmental potential of early embryos (46, 47). GSH is the most popular cellular antioxidant. It is reported that NAC can increase GSH levels (25, 48), which is consistent with our study. GSH can clear oxygen species (ROS) which are considered to promote aging, so GSH supplement could prevent oxidative stress in oocytes (23). The main reason for poor reproductive performance in women of advanced age is a high proportion of embryos aneuploidy. Cho et al. (49) discovered that aneuploidy arose from tetraploid cells due to cytokinesis failure, that ROS may be related to cytokinesis, and that cytokinesis failure was attenuated by pretreatment with NAC. It can be inferred that NAC may reduce the risk of aneuploidy. Moreover, mitochondria are closely related to the decrease of oocyte quality with age (24). Embryos from advanced age female subjects often have an abnormally high mtDNA copy number and such embryos do not recommend to transfer (50–52). However, the relationship between mtDNA integrity in oocytes and ovarian senescence is still uncertain from the qualitative point. In our present study, there was no difference in the mtDNA copy number between the two groups. Although the processing effects of NAC supplementation during IVF/ICSI cycles on aged patients were demonstrated for the first time in our study, the specific molecular mechanisms and the potential effect of NAC on oocytes and embryos remain to be further explored. We hope that the results of our present study would need more detailed research to accurately determine the ameliorative mechanism of NAC addition against the aged oocytes, and more efforts should be made to examine the molecular mechanisms of NAC actions.

The present study has certain limitations. The sample was from a single center, and most patients belonged to the same geographical area, which may have affected the results. More interventional trials are warranted to verify NAC-related clinical relevance in promoting reproductive outcomes of the subpopulations. Additionally, the study on the security of the long-term outcomes of NAC on both mother and child was overlooked.

In conclusion, we determined the major effects of NAC on improving oocyte quality through antioxidant action, leading to high-quality blastocysts by improving the GSH content. NAC pretreatment can improve the sensitivity of the ovary to exogenous gonadotropins in advanced age women, which may be helpful to promote oocyte development capacity. However, the clinical pregnancy outcomes were not affected by NAC treatment. Therefore, more clinical trials with a larger sample size are required to be designed to confirm this conclusion in future.

## Data availability statement

The original contributions presented in the study are included in the article/supplementary material, further inquiries can be directed to the corresponding author.

## Ethics statement

The studies involving human participants were reviewed and approved by Affiliated Reproductive Hospital of Shandong University. The patients/participants provided their written informed consent to participate in this study.

## Author contributions

FL contributed to the study concept and design of this study. XL, ZW, HW, and HX contributed to the acquisition, analysis, interpretation of data, and drafting of the article. YS contributed to the review and the revision of the manuscript. All authors gave final approval to this manuscript for publication.

## Funding

This study was supported by the Technology Development Program of Shandong Province (2019WS170), the National Natural Science Foundation of China (82174429), and Luo Yuankai-Zishen Yutaiwan-Research Fund for Young Scholars (20190810).

## Conflict of interest

The authors declare that the research was conducted in the absence of any commercial or financial relationships that could be construed as a potential conflict of interest.

## Publisher's note

All claims expressed in this article are solely those of the authors and do not necessarily represent those of their affiliated organizations, or those of the publisher, the editors and the reviewers. Any product that may be evaluated in this article, or claim that may be made by its manufacturer, is not guaranteed or endorsed by the publisher.
